# *Sedum guniuensis*, a New Species of *Sedum* (Crassulaceae) From Anhui, China

**DOI:** 10.1002/ece3.74120

**Published:** 2026-07-29

**Authors:** Wan‐Zeng Wang, Hai‐Jun Ma, Hong‐Ming Fang, Bai‐Lin Ding, Wei‐Yong Ni, Si‐Yu Zhang, Shi‐Hui Zou, Jian‐Wen Shao, Nai‐Wei Li, Kun Liu, Li‐Jiao Ai

**Affiliations:** ^1^ Chongqing Key Laboratory of Germplasm Innovation and Utilization of Native Plants Chongqing China; ^2^ Anhui Provincial Key Laboratory of Biodiversity Conservation and Ecological Security in the Yangte River Basin, College of Life Sciences Anhui Normal University Wuhu China; ^3^ Institute of Botany, Jiangsu Province and Chinese Academy of Sciences (Nanjing Botanical Garden Memorial Sun Yat‐Sen) Nanjing China; ^4^ Shitai Management Station of Anhui Guniujiang National Nature Reserve Chizhou China; ^5^ Shitai Forestry Bureau Chizhou China; ^6^ Qimen Management Station of Anhui Guniujiang National Nature Reserve Huangshan China

**Keywords:** Anhui, China, Crassulaceae, new species, *Sedum*

## Abstract

A new species of *Sedum* (Crassulaceae), *Sedum guniuensis* from the Guniujiang Mountains in southern Anhui, China, is described and illustrated. This new species is similar to *S. emarginatum* , but can be clearly distinguished by its creeping growth habit and smaller cordate leaves. Additionally, complete chloroplast genomes from six populations of *S. guniuensis* provide strong molecular evidence for its taxonomic separation from *S. emarginatum* .

## Graphical abstract

A new species of *Sedum* (Crassulaceae), *Sedum guniuensis* from the Guniujiang Mountains in southern Anhui, China, is described and illustrated. The new species differs from *S. emarginatum* by its smaller heart‐shaped leaves and creeping growth habit.

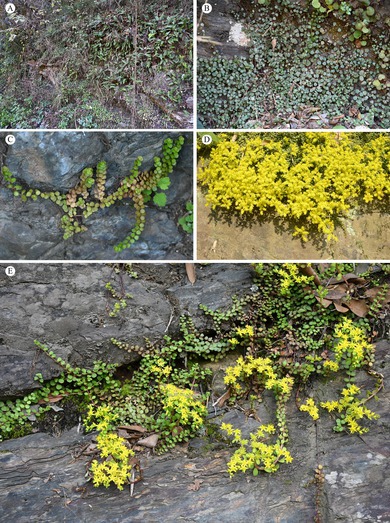

## 1 Introduction

*Sedum* Linnaeus is the largest genus in the Crassulaceae family, containing about 470 species. It is mainly found in the northern hemisphere, and some in Africa and South America in the southern hemisphere (Thiede 2007). According to the “Flora of China (Vol. 8)” there are 121 species (91 endemic) in China, mainly distributed in southwest China (Fu and Ohba 2001). In recent years, with the increase of field investigations, more and more new *Sedum* species have been discovered in China (Huang et al. 2025). Since the publication of the FOC (Vol. 8), 37 new species (including varieties) have been validly published from China, including recently published *S*. *umbraculiforme* L.J. Lu, S. Shi & Q. Fan and *S*. *taishunense* X.X. Chen, X. Liu & Y.L. Xu (She et al. 2026; Lu et al. 2026). At present, there are 158 species of *Sedum* in China, and there remains great potential for the continued discovery of new species. Due to the highly variable morphology of *Sedum* species across growth stages, high interspecific morphological similarity, as well as leaf abscission and morphological distortion after specimen preparation, species identification is difficult and prone to errors. Accurate identification requires observing their full growth cycles, ecotypes in diverse habitats and comparing microscopic anatomical features. These issues have led to long‐term neglect of field surveys, specimen collection and taxonomic research on Sedum (Zhu et al. 2024). Its species diversity remains incompletely explored, and its phylogenetic structure and interspecific relationships are still unclear (Wu et al. 2013).

During field surveys in Guniujiang Mountains of Southern Anhui, China, a new species of *Sedum* was discovered. This species resembles *S. emarginatum* , but differs significantly from it in its smaller, heart‐shaped leaves and creeping growth habit. We conducted morphological comparisons and molecular phylogenetic analysis to elucidate this putative new *Sedum* species.

## 2 Materials and Methods

### 2.1 Morphological Comparison

Specimens and individual plants of *Sedum guniuensis* were collected from wild populations for morphological comparison (Table 1). Some individuals were also transplanted to the Botanical Garden of Anhui Normal University in Wuhu for chloroplast genome analysis and further observation. Morphological data of leaf length and width of *S*. *guniuensis* and *S. emarginatum* were measured and recorded on more than 10 living individuals.

**TABLE 1:** Origins of 20 newly sequenced *Sedum*.

Population	Species	Localities	Coordinates	Altitude (m)
P1	*S. guniuensis*	Zuishanye Scenic Area, Renli Town, Shitai County, Chizhou City, Anhui Province	30.224° N/117.507° E	97
P2	*S. guniuensis*	Dashan Village, Xianyu Town, Shitai County, Chizhou City, Anhui Province	30.024° N/117.370° E	316
P3	*S. guniuensis*	Maotan Village, Qidu Town, Shitai County, Chizhou City, Anhui Province	30.214° N/117.785° E	170
P4	*S. guniuensis*	Guanglian Village, Anling Town, Qimen County, Huangshan City, Anhui Province	30.122° N/117.604° E	107
P5	*S. guniuensis*	Guniujiang Mountains, Shitai County, Chizhou City, Anhui Province	30.045° N/117.404° E	595
P6	*S. guniuensis*	Caojiawu Village, Renli Town, Shitai County, Chizhou City, Anhui Province	30.227° N/117.490° E	73
P7	*S. emarginatum*	Penglai Cave Scenic Area, Shitai County, Chizhou City, Anhui Province	30.228° N/117.545° E	92
P8	*S. emarginatum*	Jianxi Village, Biyang Town, Yi County, Huangshan City, Anhui Province	29.986° N/117.920° E	361
P9	*S. emarginatum*	Qiyun Mountain, Qiyunshan Town, Xiuning County, Huangshan City, Anhui Province	29.821° N/117.974° E	244
P10	*S. emarginatum*	Mayashu Village, Hengdu Town, Shitai County, Chizhou City, Anhui Province	30.144° N/117.600° E	91
P11	*S. emarginatum*	Guanglian Village, Anling Town, Qimen County, Huangshan City, Anhui Province	30.122° N/117.604° E	107
P12	*S. emarginatum*	Chiling, Anling Town, Qimen County, Huangshan City, Anhui Province	30.023° N/117.687° E	348
P13	*S. emarginatum*	Baishi Village, Guniujiang Scenic Area, Shitai County, Chizhou City, Anhui Province	30.085° N/117.522° E	239
P14	*S. emarginatum*	Jiadao Village, Yueyang Town, Anyue County, Ziyang City, Sichuan Province	30.093° N/105.324° E	319
P15	*S. emarginatum*	Zhonghua Village, Xiluo Subdistrict, Jinsha County, Bijie City, Guizhou Province	27.507° N/106.238° E	933
P16	*S. emarginatum*	Caodian Town, Sui County, Suizhou City, Hubei Province	32.189° N/113.674° E	128
P17	*S. emarginatum*	Anhui Normal University, Jinghu District, Wuhu City, Anhui Province	31.336° N/118.375° E	33
P18	*S. emarginatum*	Wuping Town, Jingxi City, Baise City, Guangxi Zhuang Autonomous Region	23.173° N/106.510° E	773
P19	*S. emarginatum*	Wenting Town, Yuyao City, Ningbo City, Zhejiang Province	30.031° N/121.287° E	6
P20	*S. emarginatum*	Caojiawu Village, Renli Town, Shitai County, Chizhou City, Anhui Province	30.227° N/117.490° E	73

### 2.2 Phylogenetic Reconstruction

Based on a particular combination of diagnostic characters from Table 2, it was inferred that *S. emarginatum* is the closest relative to *S*. *guniuensis* among morphologically similar species. To further conduct molecular analysis, we selected six representative individuals of *S*. *guniuensis* from distinct populations within Shitai County, Anhui Province, and 14 individuals of *S. emarginatum* from various populations for plastome sequencing: eight collected from Anhui Province and six from other provinces (Table 1). Fresh leaves of all selected samples were collected. The modified CTAB method was used to extract total DNA. Next‐generation sequencing (NGS) libraries were constructed with the VAHTS Universal DNA Library Prep Kit. High‐throughput paired‐end sequencing of 150 bp was conducted on the DNBSEQ‐T7 platform. Approximately 4 GB of raw data were generated per sample. Raw data quality was assessed using FastQC (v0.11.9), and low‐quality sequences were removed by quality control processing with fastp (v0.23.2). Plastome assembly was performed using the widely adopted software GetOrganelle. The assembled genomes were annotated using the online tool GeSeq. Additionally, plastome maps were generated via the Chloroplot web platform.

**TABLE 2:** Morphological comparisons between *S. guniuensis* and related species.

Characters	*S. guniuensis*	*S. emarginatum*	*S. jiulungshanense*	*S. makinoi*
Basal leaves	Absent	Absent	Absent	Absent
Sterile stems	Prostrate	Absent	Prostrate	Absent
Flowering stems	Prostrate, rooting at nodes	Suberect, rooting at lower nodes	Prostrate, rooting at nodes	Erect to suberect, slender to sub‐woody
Phyllotaxy	Opposite	Opposite	Whorled or uppermost opposite	Opposite
Leaf state during flowering	Opposite	Opposite	Whorled or uppermost opposite	Opposite
Leaf shape	Suborbicular to cordate, apex slightly emarginate or obtuse	Spatulate‐obovate to broadly obovate, apex emarginate	Obovate, apex obtuse or slightly emarginate	Obovate to obovate‐spatulate, base cuneate and shortly spurred, apex subacute
Spur at leaf base	Absent	Absent	Absent	Present
Leaf size (cm)	0.5–1.2 × 0.4–1.0	1–2.5 × 0.5–1.2	0.4–0.8 × 0.15–0.3	1.0–2.5 × 0.6–0.8
Inflorescence	Corymbiform, cymes usually 3‐branched	Corymbiform, cymes usually 3‐branched	Corymbiform, cymes usually 3‐branched	Corymbiform, cymes usually 2–4‐branched
Petal size (mm)	4–5 × 1–1.5	6–8 × 1.5–2	5–6 × 1–1.5	4–6 × 1–2
Sepal shape	Linear‐obovate	Lanceolate to narrowly oblong	Linear‐obovate	Linear‐spatulate, base Shortly spurred, apex Obtuse
Sepal size (mm)	2–3 × 0.6–1.2	2–5 × 0.7–2	2–3 × 1–1.5	3–4 × 0.7–1
Flowering	April–May	May–June	April–May	June–July
Fruiting	May	June	April–May	July
Distribution	China (Anhui, Zhejiang)	China (Anhui, Gansu, Hubei, Hunan, Jiangsu, Jiangxi, Shaanxi, Sichuan, Yunnan, Zhejiang)	China (Zhejiang)	Japan

To infer the phylogenetic position of the putative new species within genus *Sedum*, chloroplast genome sequences of 14 *Sedum* taxa and three outgroup species ( *Phedimus aizoon* , *Sinocrassula densirosulata*, and *Hylotelephium spectabile* ) were downloaded from NCBI. In total, 37 plastome sequences were aligned using MAFFT v.7.402 (Katoh and Standley 2013). Phylogenetic relationships were reconstructed using Maximum Likelihood (ML) method based on complete plastome data set. The best‐fit model GTR+G estimated in jModelTest was selected for Maximum Likelihood analysis. The ML tree was constructed with IQ‐TREE v.2.0.3 (Nguyen et al. 2015), with branch support assessed from 5000 replicates each of the SH approximate likelihood ratio test (SH‐aLRT) and ultrafast bootstrap (UFBS) (Hoang et al. 2018). Finally, the resulting phylogenetic trees were visualized using the online Interactive Tree Of Life (iTOL) tool, version 5 (Letunic and Bork 2021).

## 3 Results and Discussion

### 3.1 Morphology

**FIGURE 1:**
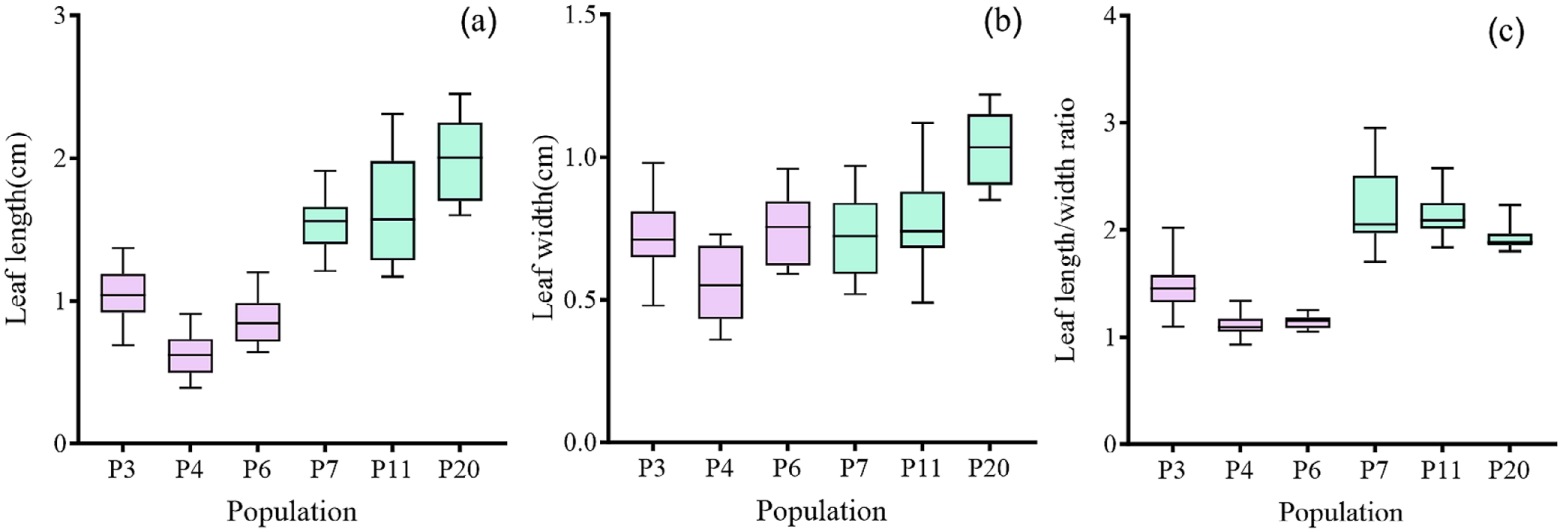
Comparison and variation of leaf length (a), leaf width (b), and their ratio (c) of *S. guniuensis* (P3, P4, P6) and *S. emarginatum* (P7, P11, P20). In the boxplot, the horizontal line indicates the median, and the bottom and top of the box represent the first and third quartiles, respectively.

*Sedum guniuensis* is morphologically similar to several species in genus *Sedum* section *Aizoon* series *Japonica*, such as *S. emarginatum* , *S. jiulungshanense*, and *S. makinoi* which are distributed in Southeast China or Japan. Although they share several common characteristics, *S. guniuensis* can be clearly distinguished from the other three species by the following key features: stems of *S. guniuensis* and *S. jiulungshanense* are prostrate, whereas those of *S. emarginatum* and *S. makinoi* are suberect; leaves of *S. guniuensis*, *S. emarginatum* , and *S. makinoi* are opposite, while those of *S. jiulungshanense* are whorled or only opposite on the upper parts of the stem; compared to *S. emarginatum* , *S. guniuensis* has distinctly smaller leaves (0.5–1.2 × 0.4–1.0 cm vs. 1–2.5 × 0.5–1.2 cm; Figure 1), petals (4–5 × 1–1.5 mm vs. 6–8 × 1.5–2 mm), and sepals (2–3 × 0.6–1.2 mm vs. 2–5 × 0.7–2 mm); leaves of *S. guniuensis* are suborbicular to cordate, obtuse or slightly emarginate versus obovate to obovate‐spatulate in *S. makinoi* . In addition, their flowering periods differ: *S. guniuensis* and *S. jiulungshanense* flower from April to May, *S. emarginatum* from May to June, and *S. makinoi* from June to July. A detailed morphological comparison is provided in the Table 2.

### 3.2 Characteristics of Plastomes and Phylogenetic Analysis

**FIGURE 2:**
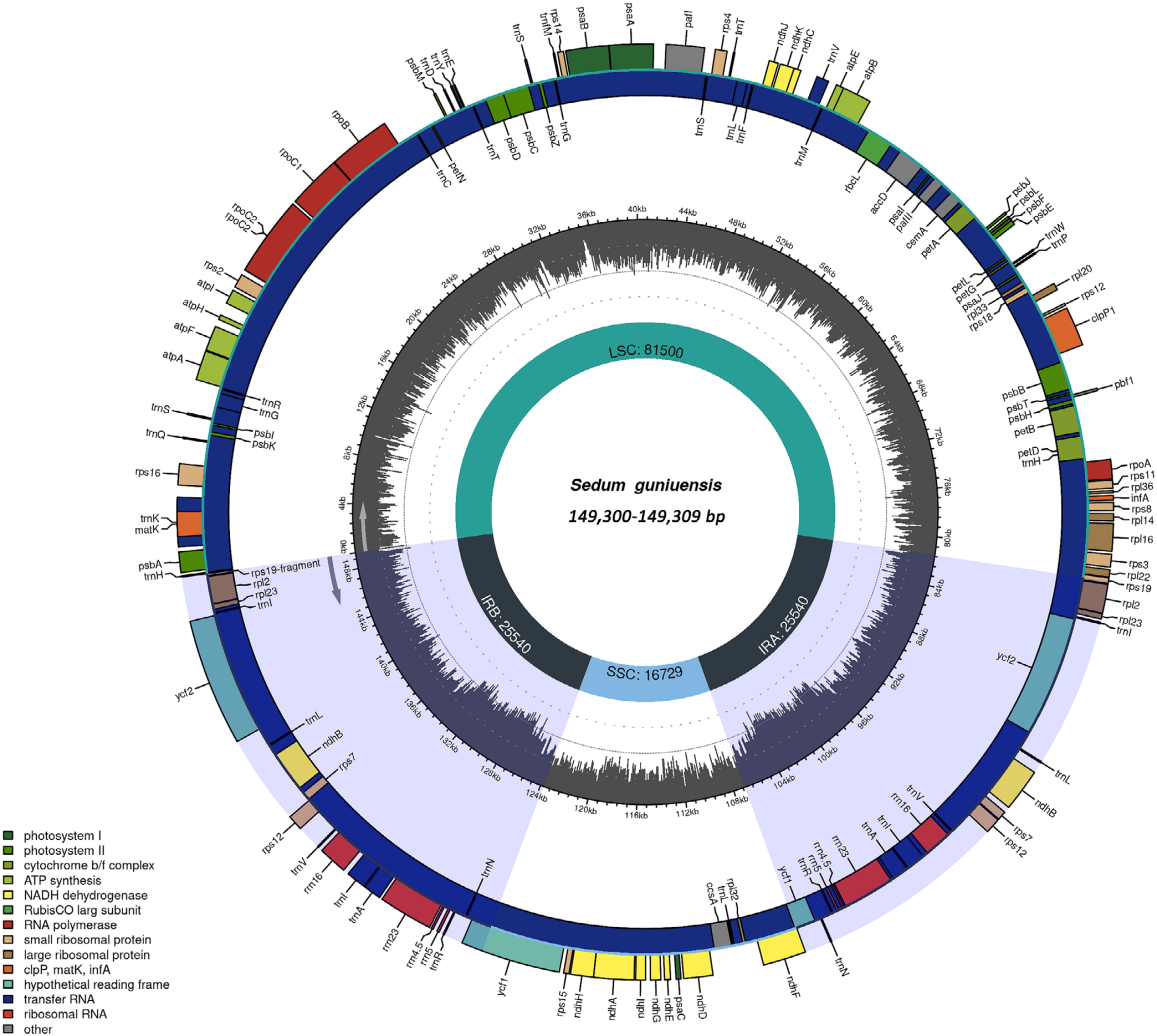
Plastome map of *Sedum guniuensis*. Genes belonging to different functional groups are color‐coded. IR, inverted repeat; LSC, large single copy; SSC, small single copy.

The plastomes of six *S. guniuensis* samples ranged in length from 149,301 to 149,309 bp (Figure 2) and exhibited a typical quadripartite structure, consisting of the large single‐copy (LSC), small single‐copy (SSC), and two inverted repeat (IRa and IRb) regions. The overall GC content was 38%. A total of 89 unique genes were annotated in the plastome, including 43 protein‐coding genes, 38 tRNA genes, and 8 rRNA genes. The plastome statistics across different populations of *S. guniuensis* are summarized in Table 3.

**TABLE 3:** Characteristics of plastomes of *Sedum guniuensis*.

Characteristic	*Sedum guniuensis*					
	P1	P2	P3	P4	P5	P6
Total length (bp)	149,301	149,309	149,309	149,300	149,309	149,308
GC%	38	38	38	38	38	38
LSC length (bp)	81,510	81,500	81,500	81,509	81,500	81,514
SSC length (bp)	16,711	16,729	16,729	16,711	16,729	16,714
IR length (bp)	25,540	25,540	25,540	25,540	25,540	25,540
Total genes	89	89	89	89	89	89
Protein‐coding genes	43	43	43	43	43	43
rRNA genes	8	8	8	8	8	8
tRNA genes	38	38	38	38	38	38

**FIGURE 3:**
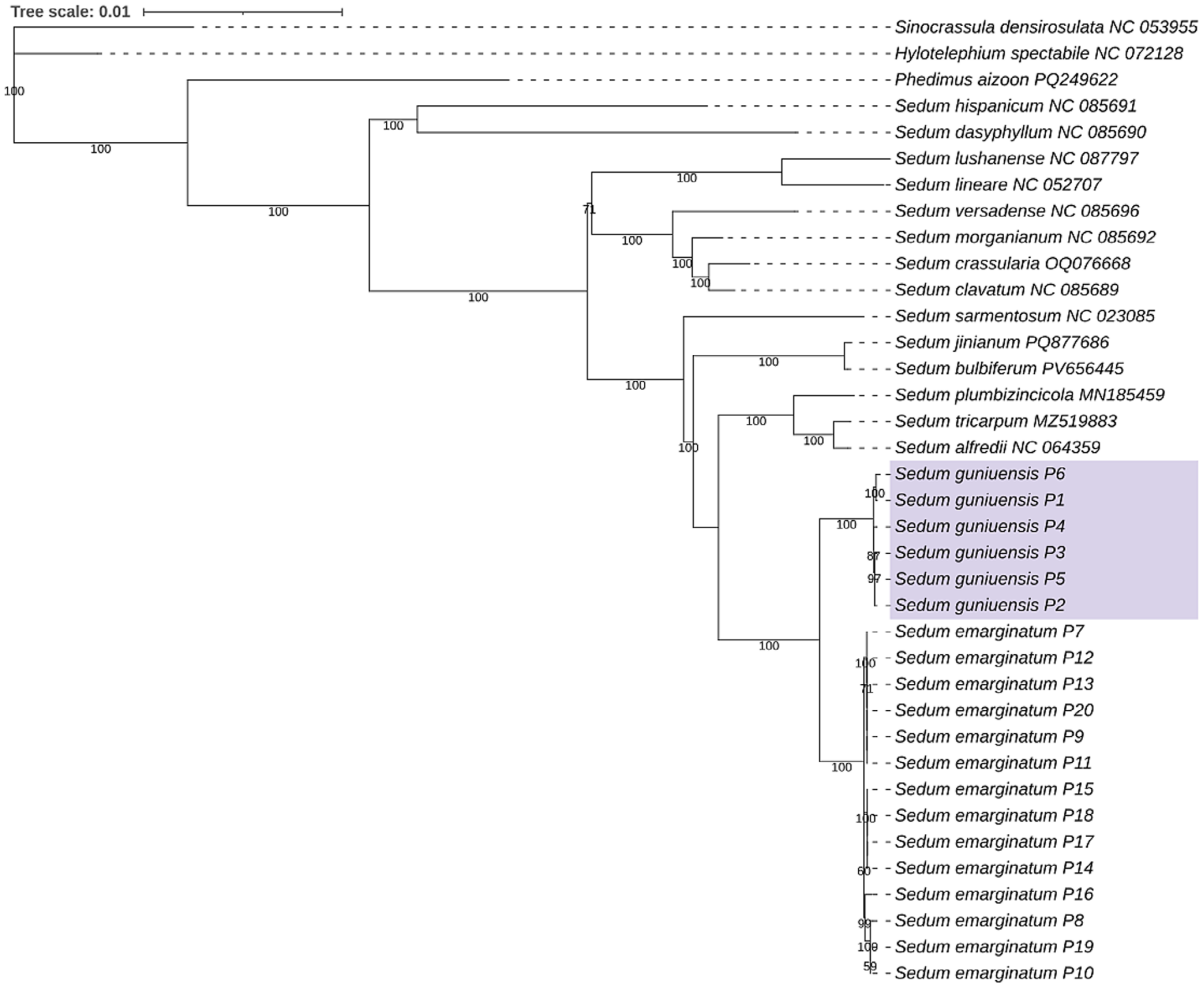
ML phylogenetic tree of *S. guniuensis* based on the complete chloroplast genome dataset. Numbers beside the nodes are bootstrap values from the ML method. Note that samples of *S. guniuensis* are marked in color.

Based on complete chloroplast genomes, we reconstructed the maximum likelihood tree (Figure 3). The maximum likelihood tree inferred from complete plastome data strongly supports *S. guniuensis* as a distinct lineage: all six accessions formed a monophyletic clade (MLBS = 100%) as sister to a well‐supported clade comprising 14 *S. emarginatum* samples from geographically distinct populations (MLBS = 100%) (Figure 2).

## 4 Taxonomic Treatment

**FIGURE 4:**
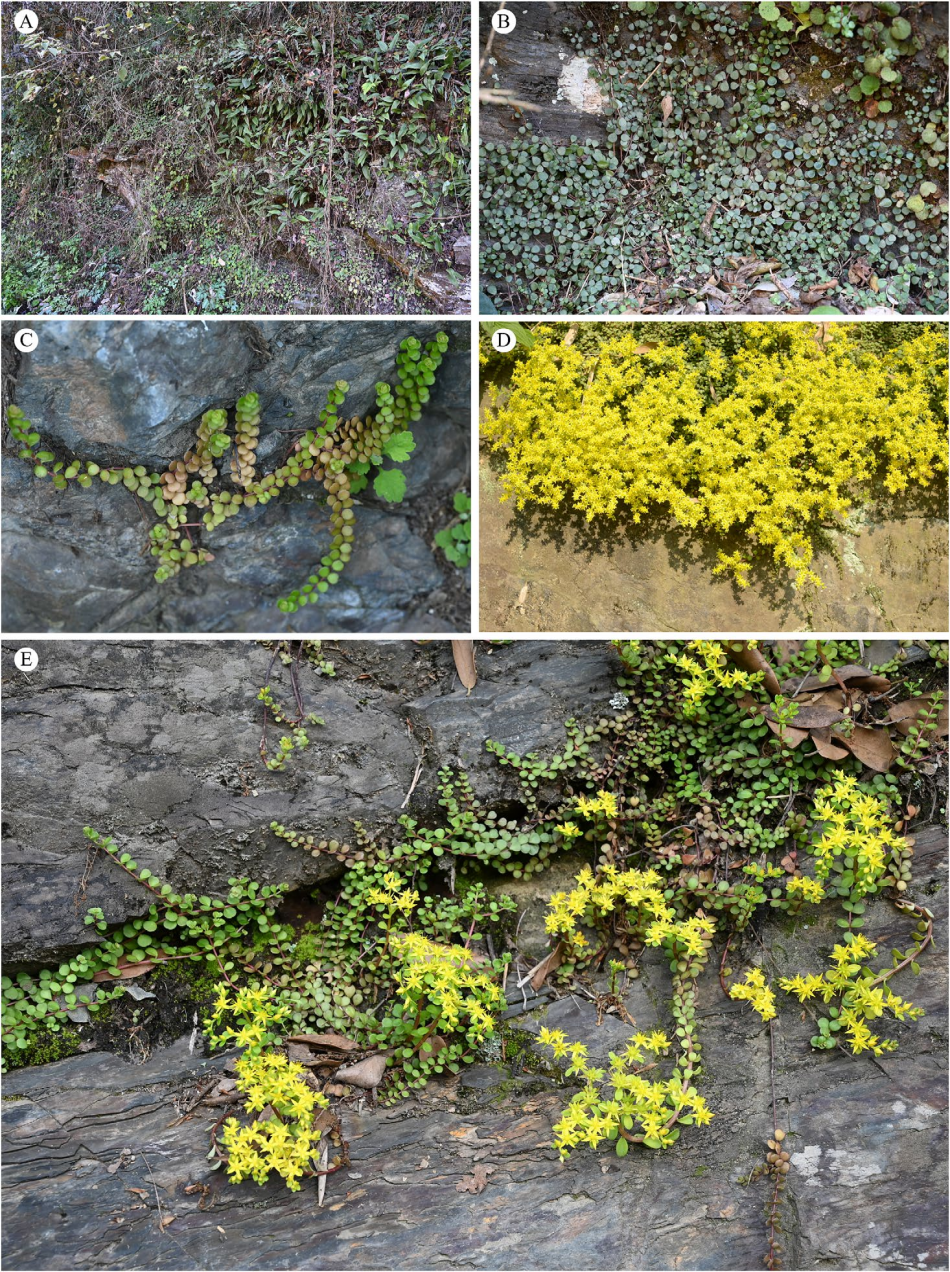
*S. guniuensis* Kun Liu, H. J. Ma & J.W. Shao, sp. nov. (A) habitat, (B) habit winter, (C) habit spring, (D) flowers, (E) plants. All are photographed by Kun Liu or Haijun Ma.

**FIGURE 5:**
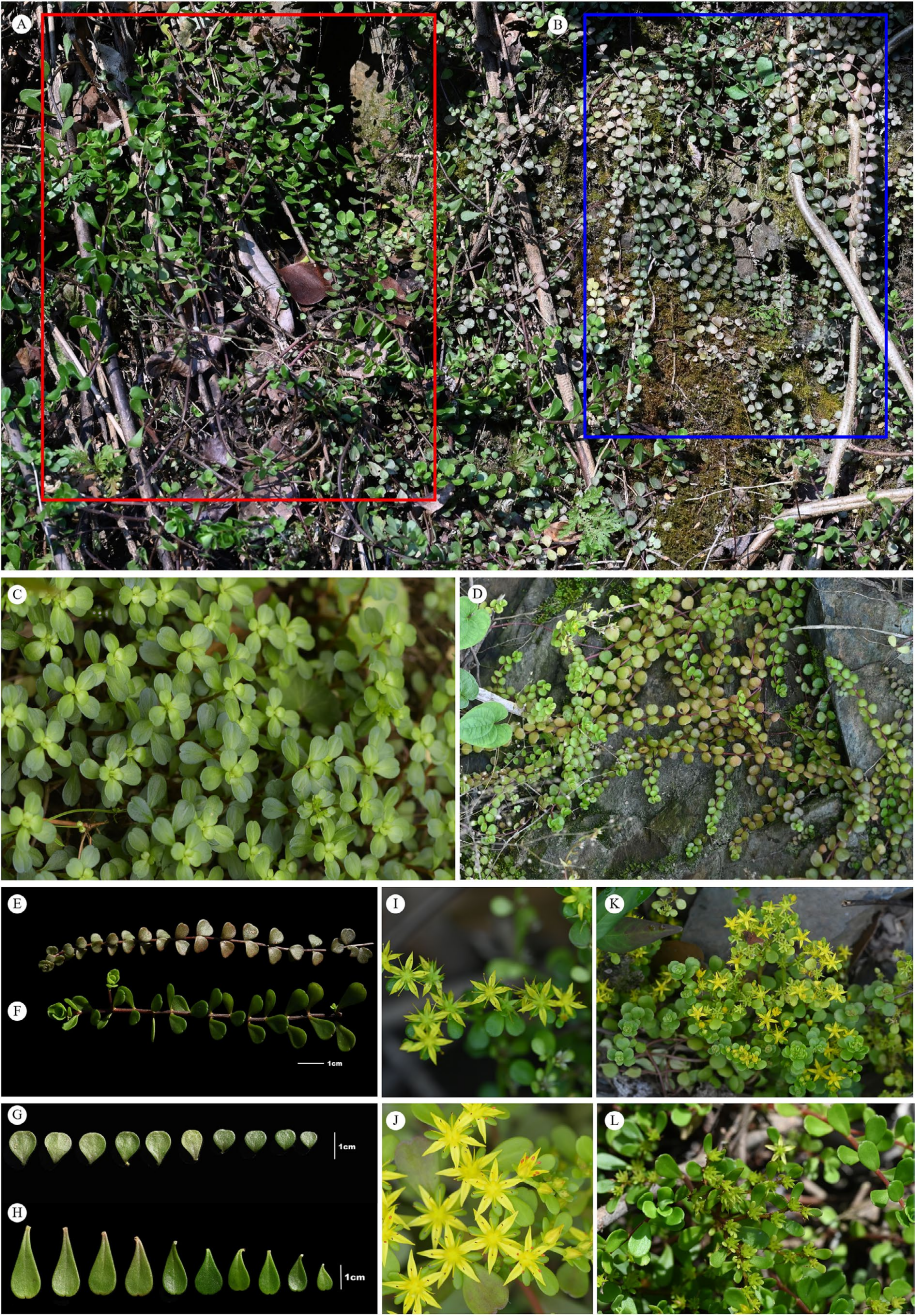
*S. guniuensis* (B, D, E, G, I, K) and *S. emarginatum* (A, C, F, H, J, L), (A, B) comparison of both species co‐occurring in sympatric communities in the wild, (C, D) plants, (E, F) cauline leaves, (G, H) leaf, (I, J) flowers, (K, L) fruits. All are photographed by Kun Liu or Haijun Ma.

*Sedum guniuensis* Kun Liu, H. J. Ma & J.W. Shao, sp. nov. (Figures 4, 5).

### 4.1 Type

CHINA. Anhui Province: Chizhou City, Shitai County, Guniujiang mountains, moist rocky cliff habitats on roadcut slopes and under broad‐leaved forests, 30.045407° N, 117.404627° E, 595 m alt., 8 May 2025, Liu K. 250508001 (fl., holotype ANUB!, isotype ANUB! NAS!).

### 4.2 Diagnosis

*Sedum guniuensis* (Figures 4 and 5) is morphologically similar to *S. emarginatum* , but differs in prostrate stems, cordate smaller leaves (0.5–1.2 × 0.4–1.0 cm vs. 1–2.5 × 0.5–1.2 cm), linear‐obovate sepals (2–5 × 0.7–2 mm), and diminutive petals (6–8 × 1.5–2 mm) (Table 2). In sympatry (Figure 4A,B), *S. guniuensis* has creeping stems and smaller leaves than *S. emarginatum* .

### 4.3 Description

Perennial herb, glabrous. Roots fibrous. Stems slender, 10–20 cm, creeping, with adventitious roots at nodes. Leaves opposite, rarely 3 whorled; blade cordate to suborbicular, 0.5–1 × 0.5–0.9 cm, apex slightly emarginate or obtuse, base cuneate, with a short spur. Flowering stems suberect, slender; leaves opposite, blade obovate, 5–10 mm long, apex obtuse, base with a short spur. Corymbose racemes, 3‐branched, 3–5 cm long, many‐flowered. Flowers sessile; bracts foliaceous, linear. Sepals 5, linear‐obovate, 1–3 mm long, apex obtuse, base with a short spur. Petals 5, yellow, ovate‐lanceolate, 4–5 × 1–1.5 mm, apex acuminate. Stamens 10, 5 long and 5 short, shorter than petals, ca. 2–3 mm long. Anthers oblong, yellow, drying brown. Scales 5, subquadrate. Carpels 5, ovate‐lanceolate, slightly divergent, ca. 6 mm long, connate at base for ca. 2 mm. Follicles divergent at maturity. Seeds castaneous, oblong‐ovate, surface papillate. Flowering in April–May, fruiting in May–June.

### 4.4 Etymology

The epithet “guniuensis” is derived from the type locality, Guniujiang mountains, Shitai County, Anhui Province, China. The Chinese name given is “Gǔ Niú Jǐng Tiān” (牯牛景天).

### 4.5 Distribution and Habitat

**FIGURE 6:**
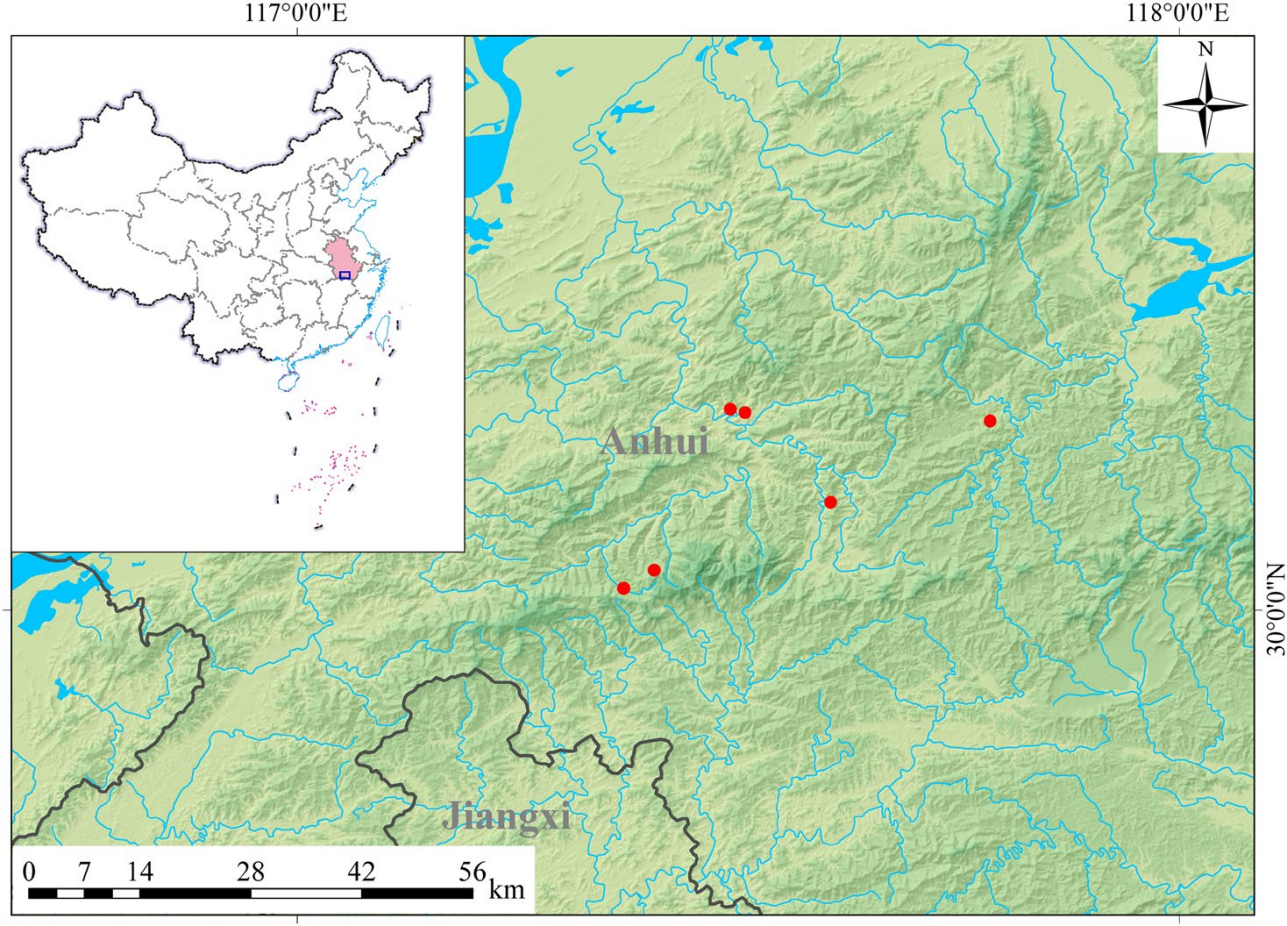
Distribution of *S. guniuensis* Kun Liu, H. J. Ma & J.W. Shao, sp. nov. ● *S. guniuensis*. The distribution points shown are based on field surveys conducted by the authors.

The new species usually grows on damp stone cliffs, 100–600 m a.s.l. It is currently found in the Guniujiang mountains in Shitai County, as well as on the other mountains surrounding the county seat of Shitai County (Figure 6). Occasionally, *S*. *guniuensis* is sympatric with *S. emarginatum* (Figure 4A,B).

### 4.6 Preliminary Conservation Status

The extent of occurrence (EOO) of *S. guniuensis* is approximately 700 km^2^ (criteria B1), indicating a very narrow distribution area. However, owing to its large population size and strong capacity for asexual reproduction and regeneration in the wild, it does not satisfy the supplementary criteria for population geographic range assessment set by the IUCN. Additionally, *S. guniuensis* is mainly distributed in the Guniujiang mountains including the Guniujiang National Nature Reserve, and the species is subject to few potential threat factors at present. Based on the IUCN Red List Categories and Criteria (IUCN Standards and Petitions Committee 2022), we classify its conservation status as Least Concern (LC).

## 5 Conclusion

In this study, we describe a new creeping species of *Sedum*, thereby expanding the genus's known diversity. Our molecular phylogenetic analyses yield critical data for resolving interspecific relationships and provide valuable insights into *Sedum* evolution. At the regional scale, this novel taxon adds an unrecognized lineage to the flora of Anhui Province and clarifies its morphological distinctions from sympatric congeners. These findings contribute to understanding the origin, distribution, and dispersal patterns of *Sedum*, and offer a foundational dataset for biodiversity assessments and habitat conservation efforts.

## Author Contributions

**Wan‐Zeng Wang:** data curation (equal), formal analysis (equal), investigation (equal), software (equal), visualization (equal), writing – original draft (equal). **Hai‐Jun Ma:** data curation (equal), investigation (equal), resources (equal), writing – original draft (equal). **Hong‐Ming Fang:** investigation (equal), resources (equal). **Bai‐Lin Ding:** investigation (equal), resources (equal). **Wei‐Yong Ni:** investigation (equal), resources (equal). **Si‐Yu Zhang:** investigation (equal), resources (equal). **Shi‐Hui Zou:** data curation (equal), methodology (equal). **Jian‐Wen Shao:** conceptualization (equal), methodology (equal), project administration (equal), supervision (equal), writing – review and editing (equal). **Nai‐Wei Li:** conceptualization (equal), methodology (equal), project administration (equal), supervision (equal), writing – review and editing (equal). **Kun Liu:** conceptualization (equal), data curation (equal), formal analysis (equal), investigation (equal), writing – original draft (equal). **Li‐Jiao Ai:** conceptualization (equal), project administration (equal), supervision (equal), writing – review and editing (equal).

## Funding

This work was supported by the Provincial Project of Science Research for Colleges and Universities of Anhui Province of China, 2023AH050150 and Chongqing Key Laboratory of Germplasm Innovation and Utilization of Native Plants, XTZW2024‐KF04.

## Ethics Statement

The authors have nothing to report.

## Conflicts of Interest

The authors declare no conflicts of interest.

## Data Availability

The raw sequence data reported in this paper have been deposited in the Genome Sequence Archive (Genomics, Proteomics & Bioinformatics 2025) in National Genomics Data Center (Nucleic Acids Res 2025), China National Center for Bioinformation/Beijing Institute of Genomics, Chinese Academy of Sciences (GSA: CRA040747) that are publicly accessible at https://ngdc.cncb.ac.cn/gsa.
